# Tuberculosis: Its Development in Childhood

**Published:** 1915-09

**Authors:** Thompson Frazer

**Affiliations:** Asheville, N. C.


### TUBERCULOSIS: ITS DEVELOPMENT IN CHILDHOOD.

BY THOMPSON FRAZER, M.D., ASHEVILLE, N. C.

Opinions as to the nature of tuberculosis have undergone radical changes within the last few years. As is well known, the disease, until recently, was regarded as an hereditary affliction; we now know that tuberculosis is only exceptionally acquired before birth, and that our former ideas were based on the frequent and early opportunities for infection which exist in tuberculous families. The fight today is waged on the principle that tuberculosis is an infectious, a communicable and a preventable disease.

"Infectious" versus "contagious." Infectious and contagious and communicable are not synonymous terms, although frequently used as if they were. By infectious we mean a disease caused by a micro-organism; for example, typhoid fever, influenza and tuberculosis are all infectious diseases. A communicable disease is one caused by a specific virus which is more or less readily transferred from one person to another. This transference may take place in a variety of ways.

Diseases differ greatly in their communicability. Yellow fever, though an infectious disease, is not readily communicable in the ordinary sense; it requires the intermedium of an insect. The more readily communicable diseases, such as measles and smallpox, are popularly known as contagious or catching. The loose use of these words has resulted in a misapprehension of the manner in which tuberculosis is acquired.

*Phthisiophobia.*—If the adjective "contagious" is applied to tuberculosis, it means the same to the general public as when it is applied to smallpox. It is no wonder that the fear of tuberculosis—or phthisiophobia—should be so widespread when we speak of the disease as "contagious." It is not contagious under ordinary conditions—that is, no momentary exposure will result in infection even indoors, while outdoors the danger may be regarded as *nil.*

*Tuberculosis a House Disease.*—While the disease is communicable and while the infection is relatively direct, we know the source of the infection, and we know how to deal with it, so that much of the apprehension existing in the minds of some people is absolutely unwarrantable and works needless hardship on the sufferer. We know that infection takes place indoors in the intimate association of family life; that tuberculosis is, indeed, a house disease.

*The Infection.*—We have also learned that the source of infection—or sources—can be controlled, theoretically, at least. We referred in our last paper to infection with bovine bacilli. This takes place through the ingestion of milk and milk products from tuberculous cows, the infectious material entering the body through some part of the digestive system. The relative importance of bovine infection has not yet been absolutely determined, but recent autopsies have tended to emphasize the infrequency of primary infection in the intestine; even in young infants the great majority of infections take place through inhalation. As Ravanel has said: "Man is the chief source of danger for man and the sputum of the consumptive plays the most important part in the dissemination of the bacilli."

*Life History of the Bacillus.*—Millions and even billions of bacilli may be excreted daily in the sputum. Fortunately, the bacillus is a strict parasite; outside the animal body it does not find conditions suitable for its existence. Nor can it reproduce its kind when thrown off from the living body. The great majority of bacilli expelled in the sputum, therefore, live but a comparatively short time. Unfortunately, however, the bacillus may retain its vitality for much longer periods, if circumstances are favorable. Thus, it was found that dried sputum from rooms occupied by consumptives was capable of producing lesions in guinea pigs after several weeks and even months. And when we consider the enormous number of bacilli that are being constantly expectorated by the consumptive—millions every day—it is not necessary that the bacilli live very long before they gain entrance into the bodies of those who live in intimate association with the consumptive. There is always a fresh source of supply at hand.

*How Infection Occurs.*—Infection, as we have said, occurs most commonly through inhaling the bacilli. This may happen in two ways—the bacilli may be inhaled, while still moist, from the air in the immediate neighborhood of the consumptive, more especially if he does not cover his mouth in the act of coughing. It has been found that during the act of coughing a fine spray is given out, containing bacilli, which float for a variable period in the air. More often the bacilli are inhaled in the form of dust derived from tuberculous sputum, which retains its virulence for a considerable period when not exposed to light. It is also possible that ingestion or swallowing of the germs, either through dust which has found its way into the food or by putting contaminated articles into the mouth, may account for other cases.

*Prevalence of Infection.*—There are, therefore, countless opportunities for infection in the neighborhood of an "open" case. And one of the most significant results of the systematic study of tuberculosis in recent years is the discovery that the prevalence of infection is much greater than we had once thought. Autopsies on persons dying from other causes reveal the fact that a large number, although never having suffered with symptoms of tuberculosis, show evidence of tuberculous infection. Percentages as high as 90 have been obtained. Even with the percentage placed at 50, it is evident that tuberculous infection is exceedingly common. The same proof of the frequency of infection was deduced as the result of the application of the cutaneous test of von Pirquet. This test—which consists in inoculating the skin with a drop of tuberculin—gives a definite "reaction," as it is called in tuberculous individuals or, more properly, in those who have been infected with the tubercle bacillus. Von Pirquet found that the reaction was uncommon in infancy, but that it increased with years, so that at the age of ten about 80 per cent. of the poorer children (on whom the test was chiefly performed) were found to be infected. Other observers have obtained somewhat lower figures, but all are agreed that the reaction is more frequent with succeeding years, while in adults a positive reaction is almost the rule.

That is to say, a large number of individuals have become infected with the tubercle bacillus, and harbor within their bodies a tuberculous focus which has not given symptoms, or which may have given symptoms, that were not recognized.

*A Child's Disease.*—In many instances the infection dates from childhood, so there is a disposition to look upon it as a child's disease, or, at any rate, as a disease due to an infection which in a great number of cases dates back to childhood. When we consider the careless habits of so many consumptives, and the exposure to which children are subjected in the home, it is not surprising that a large number of them become infected, especially in the homes of the poor, where even the most ordinary precautions are neglected.—Abstracted from *The Nurse*, by *Hygiene and the Child*.

